# Steroid profile of porcine follicular fluid and blood serum: Relation with follicular development

**DOI:** 10.14814/phy2.14320

**Published:** 2019-12-27

**Authors:** Natasja G. J. Costermans, Nicoline M. Soede, Marco Blokland, Frederike van Tricht, Jaap Keijer, Bas Kemp, Katja J. Teerds

**Affiliations:** ^1^ Human and Animal Physiology Wageningen University and Research Wageningen The Netherlands; ^2^ Adaptation Physiology Wageningen University and Research Wageningen The Netherlands; ^3^ Wageningen Food Safety Research (WFSR) Wageningen University and Research Wageningen The Netherlands

**Keywords:** follicle, follicular fluid, porcine, serum, steroid

## Abstract

The aim of this study was to identify follicular fluid (FF) steroids which reflect follicular development in the early stages of the follicular phase and to establish whether the levels of these FF steroids correspond to their levels in serum. If these relations are established, serum steroid profiles may be used to monitor follicular development already in this early stage of the follicular phase. We used samples of two experiments, one with multiparous sows at the onset of the follicular phase (weaning) and one with primiparous sows at the midfollicular phase (48 hr after weaning). Complete steroid profiles were measured in pooled FF of the 15 largest follicles and serum using high‐performance liquid chromatography‐tandem mass spectrometry. In experiment 1, pooled FF volume, as a measure for average follicle size, tended to be positively related to higher FF 17β‐estradiol levels (β = 0.56, *p* = .08). In experiment 2, a larger FF volume was related not only to FF higher 17β‐estradiol levels (β = 2.11, *p* < .001) but also to higher levels of β‐nortestosterone (β = 1.15, *p* < .0001) and its metabolite 19‐norandrostenedione (β = 1.27, *p* < .01). In addition, FF volume was related to higher FF 17α‐OH‐pregnenolone (β = 1.63, *p* = .03) and 17α‐OH‐progesterone (β = 1.83, *p* < .001), which could indicate that CYP17,20‐lyase activity is limiting for 17β‐estradiol production in larger follicles at the beginning of the follicular phase. In serum, most of the steroids were present at lower levels compared to FF, except for the corticosteroids. Serum progestins and androgens were never related to follicle pool volume and steroid levels did not differ in the midfollicular phase compared to the onset of the follicular phase in the second experiment. Serum steroid levels therefore poorly reflect the developmental stage of the follicle pool in the first half of the follicular phase of the estrous cycle in sows.

## Graphical abstract

We report complete follicular fluid (FF) and serum steroid profiles of sows in two different stages of the oestrus cycle and their relation with average follicle size. In FF, higher 17β‐estradiol, β‐nortestosterone, 19‐norandrostenedione, 17α‐OH‐pregnenolone and 17α‐OH‐progesterone concentrations were related to a larger average follicle size. Serum steroid levels poorly reflect the developmental stage of the follicle pool in the first half of the follicular phase of the oestrus cycle.

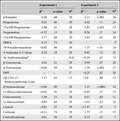

## 1 INTRODUCTION

Ovarian follicular fluid (FF) forms the microenvironment in which oocytes develop, and its composition is an important determining factor for successful oocyte development and maturation (reviewed by Li, Mckenzie, and Matzuk 2008). FF is an exudate of the blood serum and additionally contains growth factors and metabolic intermediates produced by the oocyte and somatic cells composing the follicle (Jiang et al., 2010; Sugiura, Pendola, & Eppig, 2005). Follicular somatic cells, more specifically the theca interna and mural granulosa cells, produce steroids, which are essential in sustaining oocyte and follicle development and survival and prepare the endometrium (Grupen & Armstrong, 2010; Matsuo et al., 2017; Okutsu, Itoh, Takahashi, & Ishizuka, 2010; Ting, Xu, & Stouffer, 2015). According to the two‐cell type theory of ovarian steroidogenesis, theca cells convert cholesterol into progestins and androgens, while granulosa cells express FSH‐induced aromatase (CYP19A1) to convert the androgens into 17β‐estradiol (Liu & Hsueh, 1986). This conversion already starts in early antral follicles. As follicular development continues, follicular estradiol and progesterone levels increase (Liu et al., 2000; McNatty, Hunter, Mcneilly, & Sawers, 1975; Van de Wiel, Bar‐Ami, Tsafriri, & Jong, 1983) as well as granulosa cell expression of the steroidogenic enzymes CYP19A1 and CYP17A1 (Mahajan, 2008).

Studies comparing steroid levels in FF at different stages of the estrous/menstrual cycle have mainly focused on a limited number of steroids, for example, 17β‐estradiol, testosterone, and progesterone, measured using (radio)immunoassays (e.g., McNatty et al., 1976; Van de Wiel et al., 1983; Westergaard, Christensen, & Mcnatty, 1986). Alternatively, high‐performance liquid chromatography‐tandem mass spectrometry (HPLC‐MS/MS) can be used to measure complete steroid profiles. This technique has recently been developed to accurately quantify a large number of steroids simultaneously and with high sensitivity, including their precursors and metabolites (Blokland, Tricht, Ginkel, & Sterk, 2017). Studies using HPLC‐MS/MS for FF steroid profiling have focused on FF steroid levels after controlled ovarian hyperstimulation for IVF in humans (Kushnir et al., 2009, 2016, 2012; Naessen et al., 2010; Walters et al., 2019). However, there is limited knowledge of FF steroid profiles and its relation to follicle size in early stages of the follicular phase.

Next to being released into the FF, ovarian steroids are released into the bloodstream, for instance, for transport to the brain to elicit the preovulatory LH surge to induce ovulation, while steroids from the blood may diffuse or be transported toward the follicles. FF and serum show a high correspondence in composition of small molecules and even proteins (Shalgi, Kraicer, & Soferman, 1972 and Shalgi, Kraicer, Rimon, Pinto, & Soferman, 1973). This suggests similarity between FF and blood serum steroid composition. Next to the ovaries, also the adrenal glands contribute to steroidogenesis via the production of androgen intermediates, progestins, and corticosteroids (reviewed by Rainey and Nakamura 2008), which can also influence follicular development (reviewed by Brann and Mahesh 1991). However, it is not known if FF and serum steroid composition are correlated, as the few studies that have been performed to assess these relations show conflicting results. In cycling pigs, correlations are found in the luteal phase (Naskar et al., 2016), but not in the follicular phase (Eiler & Nalbandov, 1977). Although one study in humans shows a good correlation between serum and FF steroids at oocyte retrieval after controlled ovarian hyperstimulation (Smitz, Andersen, Devroey, & Arce, 2006), a more recent study was unable to confirm these correlations (Walters et al., 2019).

If correspondence in serum and FF steroid profiles can be established or if serum steroids are related to follicular developmental stages, serum steroid profiles may be used to assess follicular development. The aim of this study was to identify FF steroids which reflect follicular development in the first half of the follicular phase of the estrous cycle and to establish whether their FF steroid levels correspond to the levels in serum. We hypothesized that FF steroid levels would correspond to follicular developmental stages and to serum steroid levels, and that serum steroid levels could therefore be used to assess follicular development in the first half of the follicular phase. To test our hypothesis, we used sows around weaning from existing studies (Costermans, Teerds, Keijer, et al., 2019 and Costermans, Teerds, Keijer, et al., 2019), as sows have a well‐defined start of the follicular phase at the end of lactation (reviewed by Soede, Langendijk, & Kemp, 2011). By performing analysis of samples from two different studies, we aimed to identify consistent and reliable relations which are present in different experimental conditions. Complete steroid profiles of porcine FF and serum were identified using HPLC‐MS/MS in order to identify their relation with follicular development and to compare FF and serum steroid levels.

## 2 MATERIALS AND METHODS

The described experiments were approved by the Animal Care and Use Committee of Wageningen University (DEC2016036 and DEC2017048) and performed according to national and EU guidelines.

### 2.1 Experiment 1

To assess relations between the steroid profiles in FF and serum and follicular development at the onset of the follicular phase, we used samples of an existing study (Costermans, Teerds, Keijer, et al., 2019), with a total of 29 multiparous N‐line sows (parity 3 to 5, Norwegian Landrace × Large White; both Topigs Norsvin). Briefly, the sows were fed a standard lactation diet (Lacto Excellent, Agrifirm) during a 26‐day lactation period and weaned on average 12.7 ± 0.1 piglets. Immediately after weaning at the onset of the follicular phase, sows were slaughtered by electrical stunning and exsanguination, after overnight fasting, to obtain the ovaries and blood samples. The blood samples were collected in 9‐ml serum clot activator tubes (Greiner Bio‐One).

### 2.2 Experiment 2

The assess relations between steroid profiles in FF and serum, and follicular development at the midfollicular phase, and to compare serum steroid profiles at the onset of the follicular phase and midfollicular phase, we included samples of a second existing study (Costermans, Teerds, Middelkoop, et al., 2019). In short, in this study we used a total of 24 primiparous TN70 sows (Topigs Norsvin) which were either full fed (6.5 kg/day, *N* = 12) or restricted fed (3.25 kg/day, *N* = 12) during the last 2 weeks of a 24‐day lactation period. Both groups were fed the same standard lactation diet (Maxima lacto, Agruniek Rijnvallei). Both groups weaned a similar number of piglets (12.4 ± 0.6 vs. 12.3 ± 0.6 piglets for full fed and restricted fed, respectively). Following overnight fasting at the time of weaning (onset of the follicular phase), sows were restrained with a nose‐sling to collect a blood sample. The sows were slaughtered by stunning and exsanguination 48 hr after weaning (midfollicular phase) to obtain the ovaries and another blood sample again after overnight fasting. Both blood samples were collected in 9‐ml serum clot activator tubes (Greiner Bio‐One).

### 2.3 Serum and follicular fluid collection

Serum tubes were kept for 1 hr at 4°C. Centrifugation took place at 3,000*g* for 10 min at 4°C and stored in −20°C until further analysis. For each sow, the left ovary was placed in plastic bags in a water bath at 37°C and the 15 largest follicles (identified upon visual inspection) were aspirated within 5 hr from collection. These 15 largest follicles were assumed to represent approximately half of the ovulatory follicle pool, as ovulation rates in modern sows are around 25–30 (da Silva et al., 2017). The FF content of the 15 largest follicles was pooled, collected in a tube, and allowed to settle for 5 min. The supernatant was removed and centrifuged at 1,900*g* for 30 min at 4°C to separate cells from the FF. The total volume of FF of the 15 largest follicles was assessed by reverse pipetting and the FF was subsequently stored at −80°C until further analysis. Pooled FF volume was used as a measure for average follicle size, as average follicle size was assessed using different methods in experiments 1 (Costermans, Teerds, Keijer, et al., 2019) and 2 (Costermans, Teerds, Middelkoop, et al., 2019). Pooled FF volume was highly correlated to average follicle size of the 15 largest follicles for both experiments (*R* ^2^ = .79 in exp 1 and *R* ^2^ = .69 in exp 2, both *p* < .0001).

### 2.4 Steroid profiling

The pooled FF of the 15 largest follicles of the left ovary and serum samples was used for profiling of endogenously produced steroid hormones. A modified UHPLC‐MS/MS method as described in Blokland et al. (2017) was used to detect steroid aglycons in follicular fluid. In short: 900 µl water was added to 100 µl follicular fluid, followed by solid‐phase extraction using an Oasis HLB 96‐well SPE plate (Waters); derivatization of aglycons was performed with picolinic acid. Chromatographic separation of the aglycons was performed using a Waters BEH C18 column (Waters) followed by analysis in a Xevo TQS mass spectrometer (Waters) in positive ESI mode.

### 2.5 Statistical analyses

All statistical analyses were performed using SAS 9.4. Normality was tested with Shapiro–Wilk tests using proc UNIVARIATE. The presence of outliers was tested by calculating the studentized residuals using proc REG and when ≥3 residuals were detected, outliers were removed from further analysis. For analysis of differences between steroid concentrations in FF and serum in multiparous sows at the onset of the follicular phase (experiment 1) and primiparous sows at the midfollicular phase (experiment 2), values were not normally distributed and could not be transformed to obtain normality. Differences in steroid concentrations were therefore analyzed by Kruskall–Wallis tests using proc NPAR1WAY. Correlations between individual steroid levels in FF and serum were tested separately for experiment 1 and experiment 2 with Spearman rank correlations using proc CORR.

Differences in serum steroid levels at the onset of the follicular phase and midfollicular phase (both experiment 2) were assessed by Tobit censored regression truncated at the lower detection limit (proc QLIM), after log transformation to obtain normality. Relations between steroid levels and amount of follicular fluid of the 15 largest follicles, as a measure for average follicle size of the pool, were also assessed by censored regression after values were log transformed to obtain normality. Relations between follicular volume and steroid levels were separately assessed for FF and serum, as many of the serum steroids were not detectable or present in very low levels. For experiment 2, effects of feed restriction on follicular fluid steroid profiles have been previously described in Costermans, Teerds, Middelkoop, et al. (2019). For the current analyses, we used models which included feed level treatment (full fed and restricted fed) and the interaction between follicle pool volume and treatment. Interactions between follicle pool volume and treatment were never significant and were therefore removed from the models. Samples of which the steroid levels were below the lower detection limit of the assay were given the value of the lower detection limit (0.01). Steroids of which less than five samples exceeded the lower detection limit of the assay were excluded from further analysis.

## 3 RESULTS

### 3.1 Relations between FF steroid levels and pooled FF volume in experiments 1 and 2

In experiment 1, average pooled FF volume was 377 ± 28 µl (123–650 µl) (Table S1). A larger pooled FF volume of the 15 largest follicles was related to higher FF 17α‐OH‐progesterone and tended to be related to higher FF17β‐estradiol levels and to higher 17α‐OH‐pregnenolone (Table 1). In experiment 2, average pooled FF volume was 280 µl (130–539 µl) for restricted fed and 427 µl (184–739 µl) for full fed (Table S1). A larger pooled FF volume was related to higher FF 17β‐estradiol, 19‐norandrostenedione, α‐testosterone, and β‐nortestosterone levels, and tended to be related to higher β‐testosterone levels. A larger pooled FF volume was also related to higher FF 17α‐OH‐pregnenolone and 17α‐OH‐progesterone levels (see Table 1).

**Table 1:** Regression coefficients (β) for the relation between follicular fluid steroid levels and follicular volume of the 15 largest follicles for experiment 1 (multiparous sows at the onset of follicular phase, *N* = 29) and experiment 2 (primiparous sows at the midfollicular phase, *N* = 24)

	Experiment 1			Experiment 2		
	β^a^	*p*‐value	N^b^	β^a^	*p*‐value	N^b^
β‐Estradiol	0.56	.08	29	2.11	<.001	24
Progesterone	0.01	.96	29	0.42	.17	24
17α‐OH‐Progesterone	1.06	.01	28	1.83	<.001	23
Pregnenolone	−0.32	.15	29	0.26	.37	24
17α‐OH‐Pregnenolone	2.77	.08	20	1.63	.03	10
DHEA	0.13	.72	29	–	–	3^c^
19‐Norandrostenedione	−0.05	.88	29	1.27	<.01	24
4‐Androsten‐3,17‐dione	0.33	.38	29	0.82	.13	23
5α‐Androstenedione^d^	–	–	N.D.	−0.25	.67	13
β‐Testosterone	0.42	.21	29	0.99	.07	24
α‐Testosterone	−0.08	.85	29	1.39	<.001	23
DHT	−	–	1^c^	−0.25	.82	20
(5β,17α)‐17‐Hydroxyandrostan‐3‐one	1.17	.65	13	−2.0	.88	13
β‐Nortestosterone	−0.06	.89	29	1.15	<.0001	24
11‐Deoxycorticosterone	−0.03	.91	29	−0.92	.21	23
Corticosterone	3.30	.13	18	0.47	.84	11
11‐Deoxycortisol	0.05	.84	29	−1.03	.22	23
Cortisol	−0.01	.97	29	−11.97	.35	5
Cortisone	−0.11	.74	29	1.75	.31	19
Cortisol/cortisone	0.01	.98	29	0.15	.43	5

N.D., not determined.

a Regression coefficients were obtained using a model with steroid concentration in follicular fluid as the dependent variable (y) and follicular fluid volume as the independent variable (x). For experiment 2, feed level (full fed or restricted fed) has been included in the model.

b Number of observations above detection limit.

c Steroids for which *N* ≤ 5 were excluded from further analysis.

d Steroid values were not determined in experiment 1.

### 3.2 Comparison of FF versus serum steroid profiles in experiments 1 and 2

FF and serum steroid profiles in experiments 1 and 2 are depicted in Table 2. Eighteen of the 19 steroids were detected in FF in both experiments (having ≥5 samples above the detection limit) and 12 of 19 and 9 of 19, in serum, respectively. In serum, all progestins, androgens, and 17β‐estradiol were either not detectable or were present at lower levels as compared to FF. In experiment 1, only 17α‐OH‐pregnenolone and 5α‐androstenedione levels were present at similar levels in FF and serum. As for the corticosteroids; for both experiments, corticosterone levels were lower, while cortisone levels were higher in FF compared to serum. Only in experiment 2, FF cortisol levels were lower compared to serum. In both experiments, FF cortisol/cortisone ratio was lower compared to serum. Correlations between individual steroids in FF and serum were never significant.

**Table 2:** Comparison of follicular fluid and serum steroid levels (ng/ml) of sows from experiment 1 (multiparous sows at the onset of follicular phase, *N* = 29) and sows from experiment 2 (primiparous sows at the midfollicular phase, *N* = 24). Values are depicted as the median (range)

	Experiment 1					Experiment 2				
	Follicular fluid		Serum		*p*‐value	Follicular fluid		Serum		*p*‐value
		N^a^		N^a^			N^a^		N^a^	
β‐Estradiol	4.1 (0.6–13.2)	29	–	2	–	4.5 (0.3–25.2)	24	–	0	–
Progesterone	12.1 (6.4–30.0)	29	1.1 (0.6–1.9)	29	<.0001	7.6 (1.4–26.0)	24	0.9 (0.6–1.5)	24	<.0001
17α‐OH‐progesterone	3.3 (0.3–12.9)	29	–	1	–	6.3 (0–64.1)	23	–	0	–
Pregnenolone	33.6 (5.6–59.9)	29	1.0 (0.1–2.4)	29	<.0001	10.4 (2.9–40.8)	24	0.1 (0–0.8)	18	<.0001
17α‐OH‐pregnenolone	0.5 (0–2.3)	20	0.1 (0–1.1)	14	.01	0 (0–4.3)	10	0 (0–2.0)	10	.99
DHEA	0.1 (0–0.4)	21	0 (0–0.5)	5	<.0001	–	3	0 (0–3.3)	10	–
19‐Norandrostenedione	1.8 (0.1–5.6)	29	–	1	–	5.7 (0.7–31.6)	24	–	1	–
4‐Androsten‐3,17‐dione	2.1 (0.7–3.6)	29	0 (0–0.3)	8	<.0001	1.6 (0–11.1)	23	–	1	–
5α‐Androstenedione^b^	–	ND	0 (0–1.6)	10	–	0.1 (0–1.3)	14	0.3 (0–1.3)	15	.57
β‐Testosterone	39.3 (4.5–120.2)	29	–	1	–	21.5 (2.9–131.0)	24	–	0	–
α‐Testosterone	3.0 (0.5–9.5)	29		7		1.6 (0–6.9)	23	–	0	–
(5β,17α)‐17‐Hydroxyandrostan‐3‐one	0 (0–2.0)	13	–	1	–	0.1 (0–3.0)	13	–	0	–
β‐Nortestosterone	3.8 (0.3–14.0)	29	–	1	–	3.5 (0.5–12.6)	24	–	0	–
11‐Deoxycorticosterone	0.8 (0.3–1.8)	29	0 (0–0.7)	11	<.0001	0.9 (0–3.0)	23	–	0	–
Corticosterone	1.1 (0–5.7)	18	1.9 (0.6–6.0)	29	.01	0 (0–0.7)	11	0.8 (0–2.3)	21	<.0001
11‐Deoxycortisol	3.0 (1.0–7.0)	29	0.8 (0–4.9)	17	<.0001	1.2 (0–5.9)	24	–	2	–
Cortisol	15.3 (4.6–42.9)	29	15.5 (0–62.8)	28	.83	0 (0–4.1)	5	7.2 (0–27.3)	20	<.0001
Cortisone	11.0 (3.2–25.9)	29	1.8 (0–6.4)	25	<.0001	2.6 (0–9.1)	19	0.8 (0–2.9)	14	<.01
Cortisol/cortisone	1.2 (0.4–5.7)	29	8.2 (1.7–28.1)	23	<.0001	0.6 (0.4–3.2)	5	3.5 (1.2–63.0)	10	.02

a Number of observations above detection limit. Steroids for which *N* ≤ 5 were excluded from further analysis.

b Steroid values were not determined for the follicular fluid in experiment 1.

### 3.3 Serum steroid profiles at the onset of the follicular phase versus midfollicular phase

Serum steroid profiles in primiparous sows (both full fed and restricted fed) at the onset of the follicular phase and at the midfollicular phase (both experiment 2) are depicted in Table 3. Serum progesterone levels tended to be higher (*p* = .06) at the midfollicular phase as compared to the onset of the follicular phase. Other steroids did not differ.

**Table 3:** Comparison of serum steroid levels (ng/ml) in primiparous sows (both full fed and restricted fed) at the onset of the follicular phase (weaning) and at the midfollicular phase (48 hr after weaning). Values are depicted as the median (range)

	Experiment 2				*p*‐value
	Onset of the follicular phase		Midfollicular phase		
		N^a^		N^a^	
Progesterone	0.9 (0.5–1.4)	24	1.0 (0.6–1.5)	24	.06
Pregnenolone	0.2 (0–1.3)	20	0.1 (0–0.8)	19	.55
17α‐OH‐pregnenolone	0 (0–3.1)	7	0 (0–2.0)	11	.26
DHEA	0 (0–4.1)	9	0 (0–3.3)	10	.65
5α‐Androstenedione	–	4	0.3 (0–1.3)	16	–
Corticosterone	0.4 (0–1.8)	18	0.8 (0–2.3)	21	.14
Cortisol	2.0 (0–21.1)	14	6.4 (0–27.3)	18	.10
Cortisone	0.9 (0–3.1)	15	0.6 (0–2.9)	14	.74
Cortisol/cortisone	1.5 (0–12.6)	8	4.1 (1.2–63.0)	9	.53

Note

For experiment 2, feed level (full fed or restricted fed) has been included in the model.

a Number of observations above detection limit. Steroids for which *N* ≤ 5 were excluded from further analysis.

### 3.4 Relations between serum steroid levels and pooled FF volume at the onset of the follicular phase (exp 1) and at the midfollicular phase (exp 2)

In serum, at the onset of the follicular phase, a larger pooled FF volume was related to higher 11‐deoxycorticosterone levels (β = 5.1, *p* = .03). No other significant relations between steroid levels and pooled FF volume have been found (Table S2).

## 4 DISCUSSION

In this study we assessed relations between FF steroid profiles and FF volume of the 15 largest follicles, as measure for average follicle size, in the first half of the follicular phase. To study this, we used samples of a study with multiparous sows at the onset of the follicular phase (Costermans, Teerds, Keijer, et al., 2019) and a study with primiparous sows at the midfollicular phase (Costermans, Teerds, Middelkoop, et al., 2019). Differences in steroid levels between the two experiments cannot be directly attributed to the different developmental stage of the follicle pool as we used sows of a different parity and different sow line, which in general show a different follicular development and ovarian steroid production (Naskar et al., 2016; van Leeuwen et al., 2011; Yun, Björkman, Oliviero, Soede, & Peltoniemi, 2018). This is the reason why the results of the two experiments were not directly compared, but rather we focused on the analysis of relations within each experiment.

In the first experiment, a larger pooled FF volume tended to be related to higher 17β‐estradiol levels. In the second experiment, pooled FF volume was not only positively related to FF 17β‐estradiol but also to FF β‐nortestosterone and its metabolite 19‐norandrostenedione. The role of these norandrogens in follicular development is not completely clear. It has, however, been suggested that they are produced as part of a minor pathway of androgen aromatization (Dehennin, Jondet, & Scholler, 1987; van Eenoo, Delbeke, Jong, & Backer, 2001) and levels are especially high in situations of high estrogen production, such as pregnancy in cows from at least 2 months before partus (de Brabander et al., 1994). Indeed, in our study, FF β‐nortestosterone and 19‐norandrostenedione were highly correlated to 17β‐estradiol levels (β = 0.65, *p* < .001 and β = 1.46, *p* < .001, respectively). This could indeed indicate that β‐nortestosterone and 19‐norandrostenedione were produced alongside 17β‐estradiol in larger follicles in the first half of the follicular phase. As follicular 17β‐estradiol levels increase with follicle size, the production of its steroid precursors increases as well. FF 17α‐OH‐pregnenolone and 17α‐OH‐progesteronelevels were higher in sows with a larger pooled FF volume. These 17α‐OH steroids can be converted into DHEA or androstenedione by CYP17A1 which catalyses 17,20‐lyase activity (reviewed by Miller & Auchus, 2011). In primiparous sows with a 18‐day lactation, CYP17,20‐lyase activity is low in the early stages of the follicular phase (3 days after weaning) and increased eightfold in theca cells of late preovulatory follicles (Corbin et al., 2003). As CYP17,20‐lyase activity was found to be low at the beginning of the follicular phase, its activity could be rate limiting for 17β‐estradiol production in sows with a larger average follicle size in the first half of the follicular phase. This rate‐limiting CYP17,20‐lyase activity may be one of the contributing mechanisms to prevent excessive androgen production at the beginning of the follicular phase. Such excessive androgen production is known to inhibit follicular growth and viability (Tarumi et al., 2012) and induces the formation of follicular cysts (Okutsu et al., 2010), similar to what is seen in patients with PCOS (Naessen et al., 2010). Other FF progestins and androgens were not related to pooled FF volume which indicates that the activity of other steroidogenic enzymes such as CYP17α‐hydroxylase, needed for the hydroxylation of pregnenolone and progesterone, and CYP19A1, needed for the conversion of androgens into estrogens, are not limiting for 17β‐estradiol production at this stage of follicular development.

In this study we also assessed if, next to FF steroid profiles, serum steroid profiles could reflect follicular development at the beginning of the follicular phase. In both of our experiments, most of the progestins, androgens, and 17β‐estradiol are either not detectable or present at very low levels in serum. In later stages of follicular development, serum contains higher levels of 17β‐estradiol which suppresses FSH secretion (Henricks, Guthrie, & Handlin, 1972; Walters et al., 2019) resulting in the selection of only those follicles which have sufficient FSH and LH receptors for ovulation (reviewed by Knox, 2005). Progestin production starts to rapidly increase in preovulatory follicles (Walters et al., 2019). This may explain the low levels of serum 17β‐estradiol and progestins in our study as serum samples were obtained from sows at the first half of the follicular phase of the cycle. Next to ovarian androgen production, the adrenal gland also contributes to androgen production, which can serve as precursors for sex steroid production (Kaminska, Opalka, Ciereszko, & Dusza, 2000). As androgen levels in serum were very low, our results suggest that the contribution of the adrenal gland to serum steroid levels is probably very limited at the first half of the follicular phase of the cycle.

We used three different methods to assess if serum steroid profiles could be used to monitor follicular developmental stage. We first established whether FF steroid levels correspond to the steroid levels in serum. In both our experiments, cortisol/cortisone was lower in follicular fluid as compared to serum, which is indicative for higher cortisol oxidation and 11β‐hydroxysteroid dehydrogenase (HSD11B) activity in follicles as compared to extragonadal tissues. As cortisol can inhibit gonadotropin‐induced follicular steroidogenesis in cultured granulosa cells (Hsueh & Erickson, 1978; Michael et al., 1993) and negatively influences in vitro oocyte maturation (Yang, Chen, & Li, 1999), it may be postulated that high follicular HSD11B activity in the porcine ovary acts as a protective mechanism to support follicular steroidogenesis and also oocyte maturation. This assumption is further supported by a study of Sunak, Green, Abeydeera, Thurston, and Michael (2007), which found increased cortisol oxidation with increasing antral follicle size, when follicular steroid production is high.

Second, to further investigate if serum steroid levels are reflective of the follicular developmental stage of the follicle pool, we assessed if serum steroid levels differ at the midfollicular phase as compared to the onset of the follicular phase in the primiparous sows. However, none of the steroids measured in serum increased from the early to the midfollicular phase, a phase corresponding to a more developed follicle pool. Finally, as a third method, we assessed if serum steroid levels were related to follicle size and found that relations were (almost) never significant. Taken together, these results show that serum steroids do not reflect follicular development in this first half of the follicular phase.

To summarize, our study describes for the first time a comparison between follicular FF and serum steroid profiles in sows at the first half of the follicular phase using results of two separate experiments. Our analysis revealed that higher follicular 17β‐estradiol production in a follicle pool with larger follicles is accompanied by β‐nortestosterone and 19‐norandrostenedione production. FF 17α‐OH‐pregnenolone and 17α‐OH‐progesterone are also related to pooled FF volume, which indicates that CYP17,20‐lyase activity is limiting for 17β‐estradiol production in larger follicles in the early follicular phase. In serum, steroids are generally present in low levels and poorly reflect the developmental stage of the follicle pool in first half of the follicular phase of the cycle.

## CONFLICT OF INTEREST

None to declare.

## AUTHOR CONTRIBUTIONS

The overall study design was performed by N. G. J. Costermans, J. Keijer, K. J. Teerds, N. M. Soede, and B. Kemp. The research was performed by N. G. J. Costermans, F. van Tricht, and M. Blokland. Analytical tools were provided by F. van Tricht and M. Blokland. All authors were involved in data interpretation. The manuscript was drafted and edited by N. G. J. Costermans, with additional editing by K. J. Teerds, J. Keijer, N. M. Soede, and B. Kemp. All authors approved the final manuscript.
