# Supplementary material for: Steroid profile of porcine follicular fluid and blood serum: Relation with follicular development
Source: Physiol Rep. 2019 Dec 27;7(24):e14320. doi: 10.14814/phy2.14320 (PMC6934872; doi:10.14814/phy2.14320)
Supplement: Supplementary file 1 [file PHY2-7-e14320-s001.docx]

|  | Experiment 1 | Experiment 2 | |
| --- | --- | --- | --- |
|  |  | Full-fed (N=12) | Restricted-fed(N=18 |
| Parity | Multiparous (3-5) | Primiparous (1) | |
| Lactation | 26 days | 24 days | |
| Lactation feed allowance | Ad libitum (N=29) | 6.5 kg/day from D10-24 | 3.25 kg/day from D10-24 |
| Body weight parturition (kg) | 247±4^1^ | 206±5 | 204±5 |
| Backfat depth parturition (mm) | 17.1±0.4 | 14.0±0.6 | 13.9±0.6 |
| Body weight weaning (kg) | 232±3 | 183±5 | 164±5 |
| Backfat depth weaning (mm) | 14.1±0.3 | 9.6±0.6 | 9.1±0.6 |
| Ovaries studied | Onset of follicular phase | Mid-follicular phase | |

**Supplemental Table S1** Overview of experiment 1 and 2

**^1^**For experiment 1, Body weight at parturition was estimated using calculations as described in Bergsma et al. (2009).
