# Supplementary material for: Steroid profile of porcine follicular fluid and blood serum: Relation with follicular development
Source: Physiol Rep. 2019 Dec 27;7(24):e14320. doi: 10.14814/phy2.14320 (PMC6934872; doi:10.14814/phy2.14320)
Supplement: Supplementary file 2 [file PHY2-7-e14320-s002.docx]

**Supplemental Table S2** Relation between serum steroid levels and follicular volume of the 15 largest follicles for experiment 1 (multiparous sows at the onset of follicular phase, N=29) and experiment 2 (primiparous sows at the mid-follicular phase, N=24).

|  | Experiment 1 | | | Experiment 2 | | |
| --- | --- | --- | --- | --- | --- | --- |
|  | β | p-value | N^2^ | β | p-value | N^2^ |
| Progesterone | 0.11 | 0.50 | 29 | 0.07 | 0.60 | 24 |
| Pregnenolone | 0.21 | 0.51 | 29 | 0.59 | 0.60 | 17 |
| 17α-OH-pregnenolone | -0.72 | 0.72 | 14 | 3.0 | 0.28 | 10 |
| DHEA | - | - | 4 | -0.38 | 0.92 | 10 |
| 5α-Androstenedione | 2.35 | 0.44 | 10 | 0.02 | 0.99 | 15 |
| 11-Deoxycorticosterone | 5.1 | 0.03 | 11 | - | - | 0 |
| Corticosterone | 0.19 | 0.56 | 29 | -0.44 | 0.67 | 21 |
| 11-Deoxycortisol | 2.69 | 0.24 | 17 | - | - | 2 |
| Cortisol | -0.42 | 0.65 | 28 | 1.95 | 0.34 | 20 |
| Cortisone | 1.49 | 0.22 | 25 | 1.18 | 0.63 | 14 |
| Cortisol/cortisone | -0.25 | 0.60 | 24 | -0.55 | 0.48 | 10 |

^1^Regression coefficients were obtained using the a model with steroid concentration in serum as the dependent variable (y) and follicular fluid volume as the independent variable (x). For experiment 2, feed level (full-fed or restricted-fed) has been included in the model ^2^Number of observations above detection limit. Steroids for which N≤5 were excluded from further analysis.
